# Effect of Pretreatment on Detection of 37 Pesticide Residues in *Chrysanthemum indicum*

**DOI:** 10.1155/2021/8854025

**Published:** 2021-12-09

**Authors:** Xiao-Ying Lu, Yan-Qin Ouyang, Wei-Ya Zeng, Cui-Qing Lin, Lu-Hua Xiao, Gui-Hua Luo, Ruo-Ting Zhan, Ping Yan

**Affiliations:** ^1^School of Pharmaceutical Sciences, Guangzhou University of Chinese Medicine, Guangzhou 510006, China; ^2^Key Laboratory of Chinese Medicinal Resources from Lingnan (Guangzhou University of Chinese Medicine), Ministry of Education, Guangzhou 510006, China; ^3^Joint Laboratory of National Engineering Research Center for the Pharmaceutics of Traditional Chinese Medicines, Guangzhou 510006, China; ^4^China Resources Sanjiu Medical & Pharmaceutical Co.,Ltd., Shenzhen 518110, China

## Abstract

This study aimed to develop a method, followed by gas chromatography-mass spectrometry, for detecting 37 pesticides in *Chrysanthemum indicum* (*C. indicum*) and investigating the decrease in the matrix-induced enhancement effect. The influence of QuEChERS extraction and matrix solid-phase dispersion (MSPD) on the recovery and matrix effect (ME) was compared. extraction and matrix solid-phase dispersion (MSPD) on the recovery and matrix effect (ME) was compared to decrease the ME. The cleanup sorbents, volume and type of solvent, and treatment time were optimized. The accuracy (as recovery), precision (as relative standard deviation, RSD), linearity, limit of quantitation, and limit of detection were determined. The recoveries at the three levels using mixed standard solution ranged between 76% and 120% with RSD ≤15%, and 76% and 120% with RSD ≤11% for MSPD and QuEChERS extraction, respectively. The results suggested that the ME for 21 pesticides was in the range of 80%–120% after MSPD and 15% after QuEChERS extraction. QuEChERS extraction was simpler and faster than MSPD. This methodology was applied in the analysis of 27 *C. indicum* samples; phorate was most frequently detected (63.0% of the sample).

## 1. Introduction

*Chrysanthemum indicum* (Ye Ju Hua) is a medicinal herb with anti-inflammatory, analgesic, antioxidant, antibiotic, and other pharmacological effects. It is widely distributed and planted in China. It is very popular in Asian countries, such as China, Korea, and Japan, as traditional medicine, herbal tea, and functional foods [1].

With the implementation of standardized cultivation of Chinese herbal medicines, many herbs, including *C. indicum*, have established planting bases. Pest control is very important to improve the yield and quality of herbs. Good agricultural practices (GAP) attach great importance to the protection of the ecological environment in the production of Chinese medicinal materials and have strict usage norms and safety standards for the control of pesticide residues, such as selecting the rational pesticide varieties, application period, and mix pesticides. During the growth period of *C. indicum*, the flowers and aerial parts of *C. indicum* can have some diseases, such as spot blight, blight, and downy mildew; the pests are mainly aphids and beetles. Omethoate, dicofol, metalaxyl, carbendazim, chlorothalonil, methyl triazine, mancozeb, carbamic acid powder, and chlordimeform are relatively common pesticides used to protect flowers and plants and maintain the production yield. The pesticides commonly used in *Chrysanthemum indicum* are low-toxic and high-efficiency pesticides, but it cannot be ruled out that their pesticides will not exceed the standard. Therefore, some residues of pesticides should remain in herbal products [2]. The residues of pesticides are closely related to the soil environment.

Pesticide residues in herbs are pesticide precursors, degradants, toxic metabolites, and impurities that remain in the medicinal parts after the use of pesticides in the growing environment, planting, processing, and storage [3]. According to the chemical structure, pesticides are divided into organophosphorus, organochlorine, pyrethroid, and carbamate. Organochlorine pesticides use benzene or cyclopentadiene as raw materials, such as hexachlorocyclohexane (BHC) and dichlorodiphenyltrichloroethane (DDT). These pesticides are chemically stable and easily accumulate in living organisms. The long-term accumulation of DDT in the human body can cause immune system dysfunction and genetic and developmental toxicity; DDT can even be carcinogenic and teratogenic. Organophosphorus pesticides are currently widely used types of pesticides, including dichlorvos, dimethoate, and phorate. Most organophosphorus pesticides have a short residual time. Hence, chronic poisoning is less. At present, the acute poisoning caused by the consumption of food containing organophosphorus pesticides ranks first in the pesticide poisoning incidents in China [4].

Pesticide residue analysis is a technique used to analyze trace components in complex mixtures. It requires high sensitivity, high recovery, good specificity and reproducibility, and easy operation. The difficulty lies in the preprocessing technology, which requires extracting the components to be tested while removing the interference components as much as possible to ensure the accuracy of the detection results and avoid instrument pollution. The pretreatment methods reported so far include the mechanical oscillation, ultrasonic extraction, solid-phase extraction (SPE) [5, 6], supercritical fluid extraction, QuEChERS (quick, easy, cheap, effective, rugged, and safe) extraction, and matrix solid-phase dispersion (MSPD) [7].

The MSPD was first reported in 1989 for the simultaneous fragmentation and extraction of the same class of components in solid and semisolid samples. MSPD includes both matrix dispersion and chromatographic separation processes [8]. The general operation is to mix and grind the sample with an SPE material, fill the obtained semi-dry state mixture in the column, and then elute with a solvent of different polarity to obtain the target compound. The method simplifies the steps in the traditional pretreatment method, such as sample homogenization, tissue cell lysis, and purification, thereby avoiding the loss. QuEChERS is a pretreatment method based on solid-liquid extraction and dispersive solid-phase extraction technology developed by Anastassiades et al. [9] and validated by Lehotay et al. [10]. Because QuEChERS is friendly, convenient, stable, and reliable, it is widely adopted by the scientific community, which can be applied to the detection of pesticide residues and the extraction of different analytes from different fields and matrices such as foods [11, 12], biological fluids [13], and environmental samples [14]. The steps of the QuEChERS method can be roughly summarized as follows: pulverizing the sample, adding the extraction solvent, adding the salt to remove the water, adding the adsorbent to remove the impurities, and detecting the supernatant. The QuEChERS method can recover alkaline components that MSPD cannot recover and can extract pesticide components with a wide range of polarities. [15, 16] Many approaches are used to detect pesticide residues, including GC, LC, GC-MS, LC-MS, and so forth [17–20]. Gas chromatography-mass spectrometry (GC-MS) not only has a high separation efficiency of gas chromatography but also the ability of mass spectrometry to accurately identify the compound structure [21–23]. It can accurately measure a small amount of various pesticide residues and corresponding metabolites in the sample at the same time. It is suitable for detecting pesticide residues in medicinal materials.

The accuracy of the trace analysis with gas chromatography (GC) is often seriously affected by ME [24]. However, in previous studies, the interactions of pesticides with the matrix were observed in the detection of pesticide residues in *C. indicum* using GC-MS. In this study, 37 types of pesticides were analyzed by GC-MS, and the MSPD and QuEChERS pretreatment methods were compared in terms of purification and ME. We aimed to establish a simple, rapid, and efficient method to analyze pesticide residues in *C. indicum*.

## 2. Materials and Methods

### 2.1. Reagents and Material

The HPLC grade waters were purified using the Milli-Q system (Millipore, USA). Acetonitrile, acetone, hexane, and ethyl acetate were obtained from Merck Company (Germany). Sorbent kits were purchased from the Agela Technologies (Tianjin, China), and QuEChERS commercial extraction bag (magnesium sulfate, NaCl 1 g, and sodium citrate dibasic) and purification bag (primary secondary amine (PSA) + graphitizing of carbon black (GCB), PSA + octadecylsilane chemically bonded silica (C_18_)) were purchased from the ANPEL Laboratory Technologies (Shanghai, China). PSA, GCB, C_18_, amino (NH_2_), silica, and florisil from the ANPEL Laboratory Technologies (Shanghai, China) were used as sorbents.

Analytical grade *α*-BHC, *γ*-BHC, heptachlor epoxide, aldrin, *β*-BHC, *δ*-BHC, *α*-endosulfan, *p*,*p*′-DDE, dieldrin, endrin, *m*,*p*′-DDD, *β*-endosulfan, endosulfan sulfate, quizalofop ethyl, and pentachloronitrobenzene were obtained from ANPEL Laboratory Technologies (Shanghai, China) (Supplementary Table 1). Alachlor, heptachlor exo-epoxide, pendimethalin, tetradifon, famphur, *o*,*o*,*o*-triethylphosphorothioate, thionazin, phorate, sulfotep, diazinon, disulfoton, dimethoate, ronnel, metalaxyl, chlorpyrifos, methyl parathion, fenthion, bromophos, parathion, quinalphos, procymidone, and profenofos were purchased from Sigma-Aldrich (Germany) (Supplementary Table 1). Each stock standard solution was prepared at various concentrations in acetone and stored in the dark at a temperature less than –20°C. Pesticides were divided into groups A and B according to their characteristics and retention time. The mixed standard working solution of each group was prepared in acetone (about 10 mg/L) and stored in the dark at a temperature less than –20°C.

### 2.2. Samples

A total of 27 *Chrysanthemum indicum* samples were collected from four hospitals (Guangzhou, China), nine pharmacies (Guangzhou, China), four medical markets (Guangzhou, China; Guangxi, China; Hebei, China; and Anhui, China), and two planting bases (Hubei, China).

### 2.3. Pretreatment Methods

The pretreatment methods were based on the previously reported methods, and the cleanup sorbent and cleanup solvent were optimized. The main steps of the MSPD method included mixing the sample with the cleanup sorbent, filling the column, extracting, concentrating, and reconstituting (Supplementary Figure 1). The main steps of QuEChERS included two parts, namely, extraction and cleanup.

### 2.4. Gas Chromatography-Mass Spectrometry Analysis

The analysis was performed using gas chromatography-mass spectrometry (GC-MS). An Agilent 7890B GC system was equipped with Agilent 5977A MSD. The chromatographic separation was performed using the Agilent DB-1701 column with a length of 30 m × ID 0.25 mm ×  and film thickness of 0.25 *μ*m. The flow of carrier gas helium was 1.3 mL/min. The oven temperature was as follows: 50°C (1 min) and 30°C min^−1^ to 160°C, 4°C min^−1^ to 200°C, 3°C min^−1^ to 230°C (2 min), 2°C min^−1^ to 250°C, 20°C min^−1^ to 270°C, and 5°C min^−1^ to 300 (5-min hold), with a total run time of 48.5 min. The injector temperature was 230°C. Injection mode was splitless mode. The MS was operated in the electron ionization mode with a transfer line temperature of 250°C and an ion source temperature of 230°C.

### 2.5. Method Validation

The method validation was performed using the following parameters, namely, accuracy (expressed as recovery), precision (expressed as RSD), linearity (expressed as *R*^2^), limit of detection (LOD), and limit of quantification (LOQ).

The accuracy was expressed as recovery. Three different levels were analyzed (0.4 mg/kg, 2 mg/kg, and 10 mg/kg), with three replicates for each level. The linearity was studied by analyzing the mixed standard solution at five concentration levels. The range of analyzed concentrations was 0.02–10.0 mg/L.

### 2.6. Matrix Effect

Initially, two pretreatment methods were evaluated in terms of matrix effect (ME) by comparison between the areas of the standard in the extract and the standard in the solvent, shown by the following equation: ME (%) = (area of the standard in the matrix/area of the standard in the solution) × 100. When the ME value is close to 100%, there are no influences by the matrix. When the ME value is out of the range 80%–120%, it means that the matrix effect is significant. [25].

### 2.7. Qualitative and Quantitative Detection

The qualitative analysis of pesticide residues referred to the method for the determination of pesticide residues (Chinese Pharmacopoeia 2015, fourth edition). During sample testing, if the retention time of the detected peak was consistent with the reference, the qualitative ions appeared in the mass spectrum after subtracting the background. Moreover, the relative abundance of the sample was consistent with the reference (relative abundance >50%, deviation allowed up to ±20%; relative abundance 20%–50%, deviation allowed ±25%; relative abundance 10%–20%, deviation allowed ±30%; and relative abundance <50%, deviation allowed ±50%). Then, the presence of the pesticide in the sample was determined. The internal standard method was used for the quantitative analysis, and the internal standard was heptachlor exo-epoxide.

## 3. Results and Discussion

### 3.1. Pesticide Selection and Grouping

A total of 37 representative pesticides were selected for analysis, including pesticides and their metabolites used in planting bases and pesticides restricted or prohibited in China and other countries, which are not easily metabolized or highly toxic. Based on the relevant literature and the actual planting situation of the base, this study summarized pesticides commonly used in the control of *C. indicum* diseases and pesticides with more domestic dosage forms; 37 pesticides were used as the detection indicators. These pesticides were divided into two groups (groups A and B) based on the following principle: (1) retention time: pesticides were grouped with close retention times and overlaps to avoid mutual interference between compounds and (2) physical and chemical properties: group A mainly included organochlorine pesticides, while group B mainly included organophosphorus pesticides (Table 1).

### 3.2. Chromatographic Analysis

**Figure 1:**
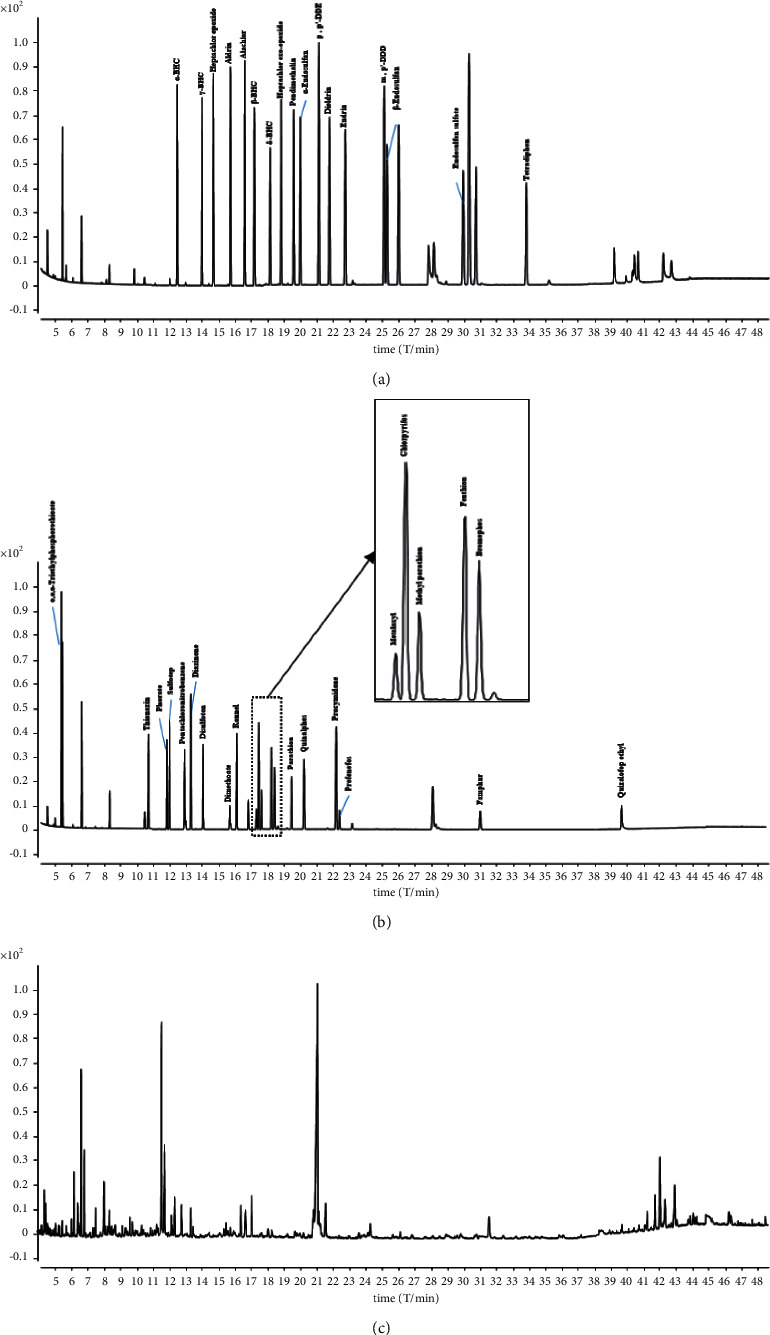
Total ion chromatograms of groups A (a), group B (b), and *C. indicum* sample (c) in the GC-MS full-scan mode.

The ions for each compound were obtained by GC-MS analysis in full-scan (Figure 1) and selective ion monitoring (SIM) modes (Table 1). The standard solutions at a concentration of 10 mg/L of pesticides were prepared in acetone. The sensitivity of some pesticide reference materials was related to chromatographic conditions. The examination of the injector temperature, transfer line temperature, and flow revealed that the injector temperature had a great influence on endosulfan sulfate, quizalofop ethyl, profenofos, and famphur.

### 3.3. Optimum Method of MSPD

**Figure 2:**
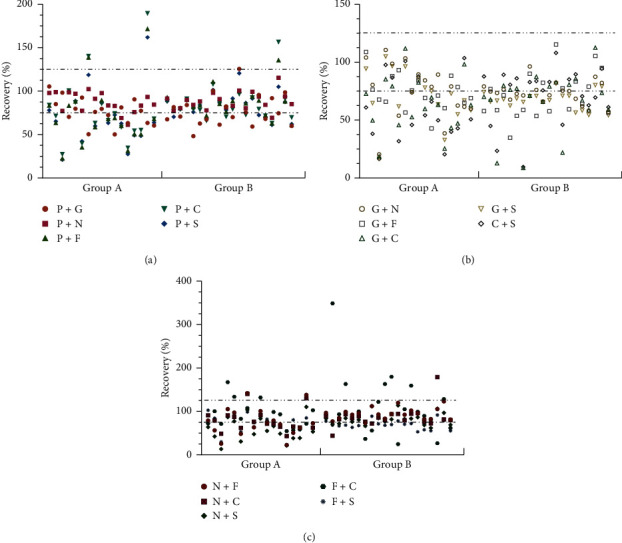
Effect of different cleanup sorbent combinations on recovery.

The MSPD processing method was as follows: 0.5 g *C. indicum* sample was grounded for 3 min in the agate mortar with 1 g of single sorbent or 0.5 g of mixed sorbents. It was filled in the column (10 mL tube, 15.8 mm × 88 mm) with anhydrous sodium sulfate at a 2 cm height of the extraction column, which was prerinsed with 4 mL of the extraction solvent. Before the liquid level reached the top of anhydrous sodium sulfate, 25 mL of extraction solvents (*n*-hexane : acetone = 4 : 6) was added to the extract. The elution solvent was concentrated to dryness, followed by the addition of 1 mL of *n*-hexane for dissolution, and filtered for GC-MS analysis. The results of the purification effect of a single sorbent on *C. indicum* indicated that GCB had a better effect on pigment removal; both GCB and C_18_ had strong adsorption on some pesticides, such as heptachlor epoxide and endrin (Figure 2). When using the NH_2_ + C_18_ mixed sorbent, the recovery of pesticides in group B was mostly in the range of 75%–125%, but the recovery of 9 of 17 pesticides in group A was less than 75%; the recovery of 2 pesticides was higher than 125% (Figure 2(c)). When using the PSA + NH_2_ mixed sorbent, the recovery of only three pesticides was less than 75% (Figure 2(a)). Therefore, PSA + NH_2_ mixed sorbent was chosen as the adsorbent filler.

**Figure 3:**
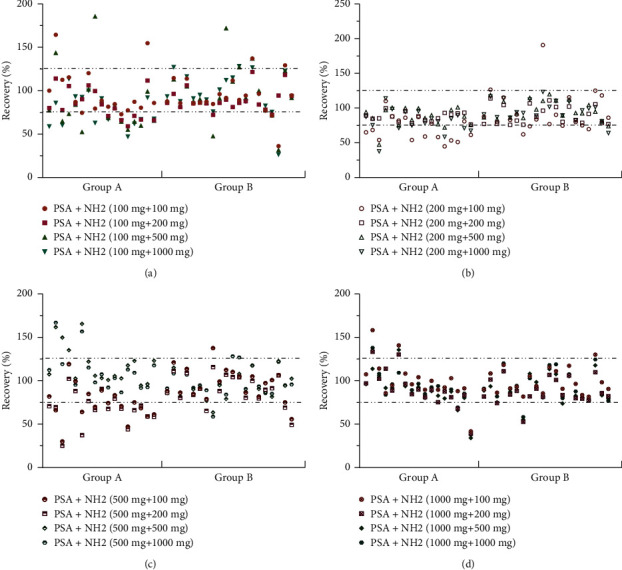
Effect of PSA and NH_2_ combination ratio on pesticide recovery.

**Figure 4:**
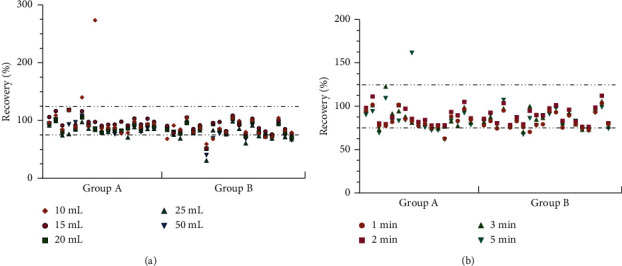
Effect of solvent volume (a) and grinding time (b) on recovery.

Furthermore, the ratio and amount of PSA and NH_2_ and the time for mixing of the cleanup sorbent with the sample powder also had a significant influence on the purification effect. Based on the recovery, a two-factor and four-level orthogonal experiment was designed to evaluate the optimal ratio of PSA and NH_2_, and the results were verified. The amount of the absorbent had a great influence on the recovery of organochlorine pesticides (group A) but had little effect on organophosphorus pesticides (group B). According to the orthogonal test results, an appropriate increase in the amount of cleanup sorbents increased the recovery; when the sorbent combination was 200 mg PSA +200 mg NH_2_, the best purification effect was achieved and verified. The recoveries of 37 pesticides ranged from 76.24% to 118.76% with RSD <10.0% (Figure 3). As shown in Figure 4(b), the repeated mixing of the adsorbent and the sample by grinding improved the extraction and purification effect. If the grinding time was too long, some pesticides, such as quizalofop ethyl, were decomposed to reduce the recovery. Overall, it was most suitable to mix the sample with the adsorbent for 2 min.

Pretreatment of pesticide residues in herbs is a process of extracting and enriching trace components. The ideal extraction solvent should be able to extract pesticides as characteristically as possible. Different types of samples have different requirements for extraction solvents; the choice of extraction solvent is a very important step in the pretreatment method. Therefore, in this study, four kinds of solvent combinations were used for extraction, which were acetonitrile (A), *n*-hexane : ethyl-acetate (9 : 1) (B), *n*-hexane : acetone (9 : 1) (C), acetone : acetonitrile (3 : 7) (D), acetone : ethyl acetate (1 : 1) (E), and *n*-hexane : acetone (4 : 6) (F). The extraction solvents A, B, and C had a large interference with *γ*-BHC (recovery >140%), while the extraction effect of B and C on dimethoate was very poor (undetectable). When using *E* as the extraction solvent, the recovery of most pesticides was less than 75%. The recovery of 16 pesticides, including heptachlor epoxide, was less than 75% using solvent combination *D*, while the extraction effect of F on dieldrin and endrin was poor. In summary, *n*-hexane : acetone (4 : 6) (F) was a suitable extraction solvent for *C. indicum* samples. The amount of the extraction solvent was also one of the factors affecting the recovery. Increasing the amount of the extraction solvent increased the recovery to a certain extent. However, excessive use not only caused unnecessary waste but also led to an increase in the number of transfers during the recovery of the solvent. In this method, 15 mL of the extraction solvent was sufficient to elute completely, and the pesticide recovery was in the range of 75%–125% (Figure 4(a)).

### 3.4. Optimum Method of QuEChERS

**Figure 5:**
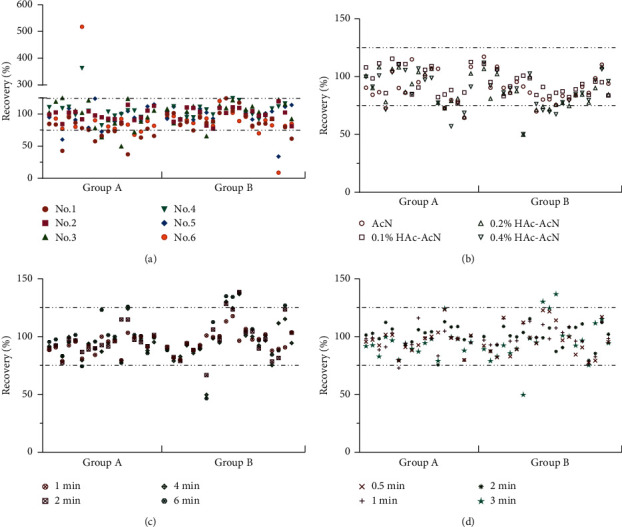
Effect of extraction conditions on pesticide recoveries in groups A and B (a) pesticide recovery for different cleanup sorbents; (b) pesticide recovery for different extraction solvents; (c) pesticide recovery for different extraction times; and (d) pesticide recovery for different purification times.

Despite the simplicity of the experiment, the effectiveness of the QuEChERS method depends on the nature of the target analyte, matrix composition, equipment, and analytical techniques available in the laboratory. Therefore, when developing the QuEChERS protocol, several parameters that affect the extraction efficiency need to be considered and optimized. [14]. The initial steps of QuEChERS extraction were as follows: 1.0 g of the sample was accurately weighed and placed in a 50 mL centrifuge tube. Then, 10 mL of the extraction solvent was precisely added and shaken at 1,000 rpm for 4 min. Furthermore, the extraction package was added, shaken at 1,000 rpm for 4 min, and centrifuged at 5,000 rpm for 5 min. The supernatant was transferred to a purification tube, shaken at 1,000 rpm for 4 min, and centrifuged at 5,000 rpm. The supernatant was filtered for GC-MS analysis. The cleanup sorbents, volume and type of solvent, and treatment time were optimized. Six kinds of cleanup sorbents were explored (Supplementary Table 2), of which No. 1, No. 5, and No. 6 had higher adsorption to profenofos (Figure 5(a)). The interference of No. 4 and No. 6 with *β*-BHC was relatively large (Figure 5(a)). When the No. 2 cleanup sorbent was used, the recovery of pesticides ranged between 82.07% and 119.95%, indicating that it had a good purification effect.

Next, the extraction effect of acetonitrile (AcN) and a solution with different concentrations of HAc (0.1%, 0.2%, and 0.4%) was investigated. Thus, 0.1% HAc was added to AcN to increase the recovery of pesticides (76.92%–115.41%). The recovery of a few pesticides, such as metalaxyl and disulfoton, reduced when the acid concentration was too high (Figure 5(b)). Different volumes (8, 10, and 15 mL) of the extraction solvent had a certain influence on the extraction effect; 10 mL was found to be sufficient.

The increase in extraction time had a great influence on the recovery of some organophosphorus pesticides. When the extraction time was 2 min, the recovery of disulfoton and methyl parathion was 66.69% and 138.47%, respectively (Figure 5(c)). The purification time had minimal effect on the recovery rate (Figure 5(d)). After adding the extraction solvent to the sample, the shaking time was 1 min. After adding the adsorbent, the same shaking was performed for 1 min.

### 3.5. Method Validation

The optimal conditions for the application of MSPD and QuEChERS to detect 37 pesticides in *C. indicum* were as follows. (1) The optimal conditions of MSPD were that 0.5 g *C. indicum* sample was taken in the agate mortar, 0.2 g PSA and 0.2 g NH_2_ were added, and the mixture was grounded for 2 min. The column was filled, followed by the addition of anhydrous sodium sulfate to an extraction column of about 2 cm height , and prerinsed with 4 mL of *n*-hexane : acetone (4 : 6). Before the liquid level reached the top of anhydrous sodium sulfate, 15 mL of *n*-hexane : acetone (4 : 6) was added to the extract, the elution solvent was concentrated to dryness, and 1 mL of *n*-hexane was added for dissolution. The filtrate was used for GC-MS analysis. (2) The optimal conditions of QuEChERS were to accurately weigh 1.0 g sample in a 50 mL centrifuge tube. Then, 10 mL of 1% HAc–AcN was added and shaken at 1,000 rpm for 1 min.

The extraction bag (4 g MgSO_4_ and 1 g NaCl) was added, shaken at 1,000 rpm for 1 min, and centrifuged at 5,000 rpm for 5 min. The supernatant was taken, transferred to a purification tube, shaken at 1,000 rpm for 1 min, and centrifuged at 5,000 rpm. The supernatant was taken and filtered for GC-MS analysis.

Different parameters, such as accuracy, precision, linearity, LOD, and LOQ, were determined (Supplementary Tables 3 and 4). The recovery and precision of the method for all pesticides at three levels (0.4 mg/kg, 2 mg/kg, and 10 mg/kg) in three replicates were determined. The recoveries at the three levels using mixed standard solution ranged between 76% and 120% with RSD ≤15%, and 76% and 120% with RSD ≤11% for MSPD and QuEChERS extraction, respectively (Supplementary Tables 3 and 4).

### 3.6. Matrix Effect

In the analysis of pesticide residues, the type and content of the matrix affect the recovery. During the gas chromatography injection process, the matrix reduces the decomposition of thermally unstable pesticides and reduces the injection of polar pesticides at the injection port. That is to say, the sample matrix increases the amount of pesticide to be analyzed that enters the column from the inlet. The calibration using pure solvent standard solutions is that ME can cause deviations in pesticide residue analysis results and recovery calculations. Several methods are used to compensate for ME, such as matrix purification. The most effective method is calibration with matrix-matched standard solutions or calibration with analytical protection agents [26, 27].

**Figure 6:**
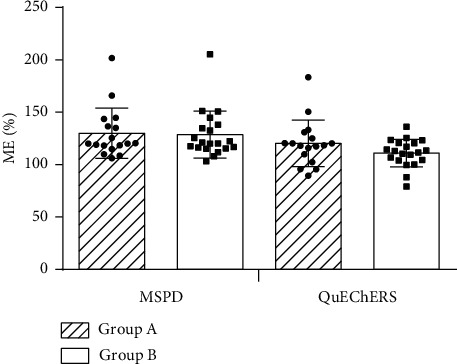
Matrix effects of different pretreatment methods.

Blank matrices of two extraction methods were prepared to compare the effects of the two pretreatment methods on ME, and a mixed standard solution was added to the matrix. The peak area of the standard in the solvent and the peak area of the matrix mixed standard were measured separately to calculate ME. After MSPD treatment, the ME value of 21 pesticides exceeded the range of 80%–120%, while after QuEChERS treatment, the ME value of only 15 pesticides exceeded 80%–120% (Figure 6).

In the *C. indicum* matrix, most of the pesticides exhibited matrix enhancement effects, especially organochlorine pesticides. MSPD and QuEChERS methods compensated the matrix enhancement effects to a certain extent using matrix-matched standard solutions, which was more obvious in the QuEChERS method.

### 3.7. Comparison of MSPD and QuEChERS

When selecting the cleanup sorbent for the MSPD treatment method, the cleanup sorbent containing GCB (including GCB + C_18_, GCB + PSA, and GCB + florisil) had a good removal effect on pigments and a strong adsorption capacity for pesticides, resulting in low recovery. Most of the improvements associated with the QuEChERS method include the optimization of amount and combinations of solvents and salts, according to the chemical nature of target analytes. These key parameters can show a relevant impact on the extraction efficiency [28]. The extraction package of the QuEChERS method was screened, revealing that the extraction package of PSA + GCB (ANPEL Laboratory Technologies) had a good purification effect. In the matrix, the most suitable cleanup sorbent for MSPD was PSA + NH_2_. This combination also had a better purification effect when used in the QuEChERS method, but the recovery of heptachlor epoxide and profenofos was less than 75%. Rejczak and Tuzimski use liquid-liquid partitioning with acetonitrile followed by dispersive solid-phase extraction cleanup using primary and secondary amine along with zirconia-coated silica particles for extraction and purification, so that the recoveries of the vast majority of the analytes were from 70 to 100% with relative standard deviations less than 20% being observed [29].

The QuEChERS method eliminated the process of drying and redissolving the solution, which not only simplified the operation but also reduced the error caused by the operation.

### 3.8. Application of *C. indicum* Sample

The qualitative and quantitative detection of pesticide residues in *C. indicum* was carried out using QuEChERS as a pretreatment method. A total of 20 pesticides were detected in 27 *C. indicum* samples, six of which were organochlorine pesticides. The detection rates of phorate and profenofos exceeded 50% (Table 2). The phorate and profenofos in the two *C. indicum* samples exceeded 2 mg/kg, and the remaining samples met the limit requirements of the Chinese Pharmacopoeia. At present, no requirements exist for pesticide residues in flowers and fruits of medicinal plants, such as *C. morifolium* Ramat, in the National Food Safety Standard–Maximum Residue Limits for Pesticides in Food (GB 2763-2016). However, it sets maximum residue limits for 50 pesticides in tea, including phorate and BHC. The phorate is a highly toxic dithiophosphate insecticide and acaricide. It inhibits cholinesterase activity and causes neurophysiological dysfunction. Profenofos is a spectral insecticide often used to control pests of cotton, vegetables, and other crops, especially for the control of resistant cotton bollworms.

## 4. Conclusions

In this study, two sample pretreatment methods (MSPD and QuEChERS) were evaluated for detecting pesticide residues in *C. indicum*, reducing the influence of matrix to improve the accuracy of detection. The results of the comparison of the two sample processing methods (MSPD and QuEChERS) show that QuEChERS is better than MSPD, but the price is also more expensive than MSPD. Compared with the extraction solvents commonly used in laboratories, such as n-hexane, acetone, and acetonitrile, acetonitrile is less toxic. QuEChERS method selects acetonitrile as the extraction solvent. Adding acetic acid according to the nature of the sample is beneficial to the recovery of pesticides. Anhydrous magnesium sulfate has a strong dehydration capacity, which can meet the rapid dehydration needs of QuEChERS. PSA can absorb impurities such as sugars and fatty acids in the sample to achieve the purpose of purification. Both methods have their own advantages. If we want to apply the method to actual sample detection, we need a simpler and faster method. A GC-MS method for detecting pesticide residues in *C. indicum* was developed. The accuracy, precision, linearity, and LOQ results showed that the proposed method was feasible and applicable to detect 37 pesticide residues in *C. indicum* samples. The calibration using matrix-matched standard solutions effectively compensated for the ME of pesticide residue analysis in *C. indicum*. As an important food and dual-use product, *C. indicum* is widely used by consumers in their daily lives, especially in China. Therefore, it is necessary to establish a faster and more effective method for analyzing pesticide residues in *C. indicum*, a matrix to ensure a healthy product to its consumers.

## Figures and Tables

**Table 1:** Qualitative and quantitative ions of group A and group B pesticides.

Group A						Group B					
Name	Retention time (T/min)	Quantitative ion	Qualitative ions			Name	Retention time (T/min)	Quantitative ion	Qualitative ions		
*α*-BHC	12.446	181	183	217	219	*o*,*o*,*o*-Triethylphosphorothioate	5.366	198	121	97	93
*γ*-BHC	13.971	181	183	111	219	Thionazin	10.668	97	96	107	143
Heptachlor epoxide	14.664	100	272	274	270	Phorate	11.82	75	121	97	260
Aldrin	15.69	66	263	91	265	Sulfotep	11.973	322	97	202	
Alachlor	16.575	45	160	188	146	Pentachloronitrobenzene	12.912	267	142	214	249
*β*-BHC	17.175	181	183	219	109	Diazinon	13.305	137	179	152	199
*δ*-BHC	18.121	181	183	219	217	Disulfoton	14.058	88	89	97	
Heptachlor exo-epoxide	18.98	253	255	81	351	Dimethoate	15.676	87	93	125	79
Pendimethalin	19.586	252	162	253	281	Ronnel	16.116	285	287	125	109
*α*-Endosulfan	19.972	195	241	237	239	Metalaxyl	17.355	206	132	160	146
*p*,*p*′-DDE	21.111	246	318	248	316	Chlorpyrifos	17.481	197	97	199	314
Dieldrin	21.757	79	81	82	263	Methyl parathion	17.661	109	125	263	
Endrin	22.743	263	81	265	261	Fenthion	18.287	278	125	109	169
*m*,*p*′-DDD	25.141	235	237	165		Bromophos	18.467	331	329	125	333
*β*-Endosulfan	25.301	195	237	207	241	Heptachlor exo-epoxide	18.985	253	255	81	351
Endosulfan sulfate	29.937	123	272	183	237	Parathion	19.456	109	97	291	139
Tetradifon	33.773	159	111	227	229	Quinalphos	20.232	146	157	118	156
						Procymidone	22.239	96	283	67	285
						Profenofos	22.46	139	97	207	206
						Famphur	30.968	218	125	93	217
						Quizalofop ethyl	39.729	299	372	163	243

**Table 2:** Detection rates of pesticides in 27 *Chrysanthemum indicum* samples (∗ indicates that the limit is exceeded).

Name	Detection rate/%	Content/mg·kg^−1^
Phorate	62.96	
Profenofos	59.26	
Aldrin and dieldrin	47.04	2.7^*∗*^
*o*,*o*,*o*-Triethylphosphorothioate	33.33	
Dimethoate	29.63	
Thionazin	14.81	
Methyl parathion	14.81	
Procymidone	14.81	
Fenthion	11.11	
BHC	11.11	
Disulfoton	11.11	
Pentachloronitrobenzene	7.41	
Alachlor	7.41	
Endrin	7.41	
Sulfotep	7.41	
Quinalphos	3.7	
Pendimethalin	3.7	
Diazinon	3.7	
Parathion	3.7	

## Data Availability

All the data used during the study appear in the submitted article.

## Supplementary Materials

Supplementary Materials Some figures and tables are included in the supplementary file.
