# Supplementary material for: Effect of Pretreatment on Detection of 37 Pesticide Residues in Chrysanthemum indicum
Source: J Anal Methods Chem. 2021 Dec 9;2021:8854025. doi: 10.1155/2021/8854025 (PMC8677409; doi:10.1155/2021/8854025)
Supplement: Supplementary Materials — Some figures and tables are included in the supplementary file. [file 8854025.f1.zip › 8854025.f1/Table 1. Information of pesticide standards..docx]

Table 1. Information of pesticide standards.

| No. | Name | Fineness | Company |
| --- | --- | --- | --- |
| 1 | α-BHC | 100mg/mL in N-hexane | ANPEL Laboratory Technologies |
| 2 | γ-BHC | 100mg/mL in N-hexane |  |
| 3 | Heptachlor epoxide | 100mg/mL in N-hexane |  |
| 4 | Aldrin | 100mg/mL in N-hexane |  |
| 5 | β-BHC | 100mg/mL in acetone |  |
| 6 | δ-BHC | 100mg/mL in N-hexane |  |
| 7 | α-Endosulfan | 100mg/mL in acetone |  |
| 8 | p,p'-DDE | 100mg/mL in N-hexane |  |
| 9 | Dieldrin | 100mg/mL in N-hexane |  |
| 10 | Endrin | 100mg/mL in N-hexane |  |
| 11 | m,p'-DDD | 100mg/mL in N-hexane |  |
| 12 | β-Endosulfan | 99.6 |  |
| 13 | Endosulfan sulfate | 96.6 |  |
| 14 | Quizalofop ethyl | 100mg/mL in acetone |  |
| 15 | Pentachloronitrobenzene | 100mg/mL in N-hexane |  |
| 16 | Alachlor | 99.8 | Sigma–Aldrich |
| 17 | Heptachlor exo-epoxide | 98.7 |  |
| 18 | Pendimethalin | 98.8 |  |
| 19 | Tetradiphon | 99.1 |  |
| 20 | o,o,o-Triethylphosphorothioate | 2000 μg/mL in hexane: acetone (80:20) |  |
| 21 | Thionazin | 99.0 |  |
| 22 | Phorate | 95.0 |  |
| 23 | Sulfotep | 95.0 |  |
| 24 | Diazinone | 98.0 |  |
| 25 | Disulfoton | 98.0 |  |
| 26 | Dimethoate | 99.5 |  |
| 27 | Ronnel | 98.0 |  |
| 28 | Metalaxyl | 98.0 |  |
| 29 | Chlorpyrifos | 99.7 |  |
| 30 | Methyl parathion | 1000 μg/mL in acetone |  |
| 31 | Fenthion | 97.3 |  |
| 32 | Bromophos | 99.5 |  |
| 33 | Parathion | 99.0 |  |
| 34 | Quinalphos | 99.3 |  |
| 35 | Procymidone | 99.9 |  |
| 36 | Profenofos | 96.6 |  |
| 37 | Famphur | 98.0 |  |
