# Supplementary material for: Effect of Pretreatment on Detection of 37 Pesticide Residues in Chrysanthemum indicum
Source: J Anal Methods Chem. 2021 Dec 9;2021:8854025. doi: 10.1155/2021/8854025 (PMC8677409; doi:10.1155/2021/8854025)
Supplement: Supplementary Materials — Some figures and tables are included in the supplementary file. [file 8854025.f1.zip › 8854025.f1/Table 2. Cleanup sorbent for QuEChERS extraction.docx]

Table 2. Cleanup sorbent for QuEChERS extraction

| No. | Composition |
| --- | --- |
| 1 | PSA + GCB (Agela Technologies) |
| 2 | PSA + GCB (ANPEL Laboratory Technologies) |
| 3 | PSA + C_18_ (ANPEL Laboratory Technologies) |
| 4 | PSA |
| 5 | PSA + NH_2_ |
| 6 | PSA + GCB |
