# Supplementary material for: Effect of Pretreatment on Detection of 37 Pesticide Residues in Chrysanthemum indicum
Source: J Anal Methods Chem. 2021 Dec 9;2021:8854025. doi: 10.1155/2021/8854025 (PMC8677409; doi:10.1155/2021/8854025)
Supplement: Supplementary Materials — Some figures and tables are included in the supplementary file. [file 8854025.f1.zip › 8854025.f1/Table 3. Linearity (expressed as R2), limit of quantification (LOQ), and three levels of accuracy of MSPD methods.docx]

| Table 3. Linearity (expressed as R2), limit of quantification (LOQ), and three levels of accuracy (expressed as recovery; *: 0.4 mg/kg, **: 2 mg/kg, ***:10mg/kg) of MSPD methods | | | | | | | | | | | | | | |
| --- | --- | --- | --- | --- | --- | --- | --- | --- | --- | --- | --- | --- | --- | --- |
| Name | Regression equation | R2 | 95% confidence interval of slope | | 95% confidence interval of intercept | | LOD(mg/kg) | LOQ(mg/kg) | * Recovery/% | * RSD/% | ** Recovery/% | ** RSD/% | *** Recovery/% | *** RSD/% |
|  |  |  | toplimit | lower limit | toplimit | lower limit |  |  |  |  |  |  |  |  |
| α-BHC | y = 82.2x - 315 | 0.997 | 75.4 | 89.0 | -809 | 179 | 0.0488 | 0.163 | 116 | 3.92 | 97.3 | 2.86 | 104 | 1.84 |
| γ-BHC | y = 88.5x - 351 | 0.999 | 86.6 | 90.4 | -761 | 59.4 | 0.0894 | 0.298 | 90.5 | 5.70 | 105 | 3.06 | 83.7 | 3.28 |
| Heptachlor epoxide | y = 75.9x - 504 | 0.999 | 73.2 | 78.6 | -1196 | 188 | 0.188 | 0.626 | 82.9 | 6.92 | 91.0 | 8.29 | 98.2 | 8.22 |
| Aldrin | y = 62.8x - 203 | 1.00 | 61.8 | 63.7 | -462 | 55.3 | 0.0708 | 0.236 | 83.1 | 7.45 | 89.7 | 4.68 | 100 | 1.14 |
| Alachlor | y = 86.2x - 504 | 1.00 | 84.4 | 87.9 | -997 | -42.1 | 0.0324 | 0.108 | 84.4 | 2.51 | 87.1 | 2.58 | 96.8 | 2.61 |
| β-BHC | y = 101x - 706 | 0.999 | 98.9 | 103 | -1153 | -259 | 0.0898 | 0.300 | 82.0 | 1.18 | 107 | 3.72 | 90.5 | 1.93 |
| δ-BHC | y = 68.0x - 322 | 1.00 | 67.0 | 68.9 | -541 | -105 | 0.0986 | 0.329 | 114 | 3.48 | 96.9 | 4.60 | 100 | 3.56 |
| Heptachlor exo-epoxide | y = 24.7x - 135 | 0.999 | 24.2 | 25.2 | -243 | -27.3 | 0.281 | 0.936 | 87.9 | 1.63 | 85.0 | 1.53 | 89.0 | 6.21 |
| Pendimethalin | y = 22.2x - 180 | 0.999 | 21.7 | 22.7 | -279 | -82.0 | 0.0808 | 0.270 | 112 | 2.93 | 89.3 | 6.37 | 101 | 4.11 |
| α-Endosulfan | y = 30.3x - 137 | 0.999 | 29.7 | 30.9 | -237 | -37.8 | 0.269 | 0.898 | 88.0 | 6.35 | 77.0 | 3.63 | 95.4 | 2.09 |
| p,p'-DDE | y = 219x - 1547 | 0.999 | 211 | 227 | -3572 | 477 | 0.0428 | 0.143 | 87.4 | 2.97 | 77.0 | 4.36 | 96.7 | 2.18 |
| Dieldrin | y = 74.1x - 328 | 0.999 | 72.7 | 75.4 | -562 | -93.8 | 0.0824 | 0.275 | 98.0 | 3.22 | 78.6 | 2.77 | 87.2 | 5.32 |
| Endrin | y = 31.9x - 266 | 0.999 | 30.6 | 33.2 | -619 | 86.7 | 0.355 | 1.18 | 98.7 | 7.72 | 77.9 | 0.95 | 76.7 | 8.11 |
| m,p'-DDD | y = 168.63x - 770.92 | 1.00 | 166 | 171 | -1476 | -66.1 | 0.0370 | 0.123 | 118 | 3.36 | 106 | 5.07 | 111 | 4.72 |
| β-Endosulfan | y = 34.8x - 316.7 | 0.999 | 33.3 | 36.3 | -717 | 83.4 | 0.359 | 1.20 | 82.1 | 3.36 | 82.6 | 5.23 | 79.0 | 6.29 |
| Endosulfan sulfate | y = 13.502x - 377.05 | 1.00 | 13.04 | 13.96 | -515 | -239 | 0.968 | 3.23 | 90.4 | 2.47 | 109 | 1.58 | 92.6 | 10.1 |
| Tetradiphon | y = 59.8x - 544 | 0.999 | 58.3 | 61.3 | 907 | 182 | 0.151 | 0.506 | 118 | 4.15 | 93.5 | 4.73 | 113 | 5.62 |
| o,o,o-Triethylphosphorothioate | y = 123511x - 1277 | 0.999 | 121847 | 125177 | -2142 | -416 | 0.0208 | 0.0690 | 109 | 2.46 | 89.3 | 3.92 | 100 | 5.80 |
| Thionazin | y = 74105x - 4590 | 0.999 | 71815 | 76394 | -6903 | -2279 | 0.0102 | 0.0340 | 105 | 8.45 | 101 | 10.3 | 103 | 2.51 |
| Phorate | y = 94825x - 4706 | 0.999 | 92189 | 97460 | -6653 | -97461 | 0.0242 | 0.0806 | 84.4 | 5.46 | 83.7 | 6.80 | 94.9 | 5.06 |
| Sulfotep | y = 82384x - 9464 | 0.999 | 80698 | 84070 | -12817 | -6113 | 0.0404 | 0.134 | 93.8 | 3.93 | 95.9 | 5.81 | 111 | 5.46 |
| Pentachloronitrobenzene | y = 33899x - 4071 | 0.999 | 33145 | 34653 | -5286 | -2856 | 0.105 | 0.350 | 91.5 | 2.98 | 90.1 | 4.97 | 112 | 1.89 |
| Diazinone | y = 94663x - 18330 | 0.999 | 91775 | 97552 | -25678 | -10983 | 0.0404 | 0.135 | 84.7 | 6.16 | 103 | 4.32 | 99.0 | 3.01 |
| Disulfoton | y = 81912x - 5166 | 0.999 | 79020 | 84803 | -8080 | -2254 | 0.0300 | 0.0998 | 103 | 2.97 | 87.7 | 4.13 | 92.0 | 1.05 |
| Dimethoate | y = 19628x - 2423 | 0.999 | 18827 | 20429 | -3940 | -907 | 0.0490 | 0.164 | 76.6 | 1.87 | 93.5 | 5.79 | 94.7 | 6.27 |
| Ronnel | y = 203392x - 36598 | 0.999 | 199749 | 207036 | -43163 | -30036 | 0.0400 | 0.133 | 94.0 | 4.23 | 86.1 | 7.22 | 97.0 | 3.36 |
| Metalaxyl | y = 12141x - 1498 | 1.00 | 11930 | 12353 | -1834 | -1162 | 0.0570 | 0.190 | 120 | 9.57 | 95.2 | 9.69 | 118 | 0.51 |
| Chlorpyrifos | y = 78127x - 5694 | 0.999 | 75933 | 80326 | -7405 | -3986 | 0.0388 | 0.129 | 114 | 9.27 | 107.9 | 6.42 | 110 | 0.99 |
| Methyl parathion | y = 11906x - 1440 | 0.999 | 11431 | 12381 | -1976 | -905 | 0.0542 | 0.181 | 81.7 | 9.30 | 80.2 | 9.85 | 96.3 | 6.43 |
| Fenthion | y = 143006x - 28484 | 0.999 | 136828 | 149183 | -42916 | -14059 | 0.0428 | 0.143 | 97.0 | 4.89 | 85.3 | 4.73 | 99.8 | 2.03 |
| Bromophos | y = 118908x - 15315 | 0.999 | 116097 | 121716 | -19289 | -11341 | 0.0630 | 0.210 | 86.4 | 13.1 | 111 | 0.47 | 101 | 1.82 |
| Parathion | y = 22892x - 1071 | 0.9991 | 22269 | 23516 | -1428 | -715 | 0.0660 | 0.220 | 94.2 | 6.06 | 87.4 | 2.42 | 101 | 1.68 |
| Quinalphos | y = 83057x - 10805 | 0.999 | 80933 | 85181 | -13862 | -7749 | 0.0620 | 0.207 | 82.6 | 8.87 | 83.2 | 4.77 | 96.9 | 2.35 |
| Procymidone | y = 235173x - 18846 | 0.999 | 231839 | 238511 | -23596 | -14102 | 0.0414 | 0.138 | 89.6 | 7.71 | 91.6 | 4.56 | 88.9 | 1.06 |
| Profenofos | y = 3658x - 210 | 0.999 | 3454 | 3864 | -503 | 82 | 0.284 | 0.945 | 94.1 | 7.95 | 77.4 | 8.90 | 93.8 | 3.39 |
| Famphur | y = 20972x - 1487 | 0.999 | 20452 | 21492 | -2136 | -840 | 0.163 | 0.543 | 115 | 2.41 | 101 | 1.73 | 102 | 3.54 |
| Quizalofop ethyl | y = 20480x - 2247 | 0.999 | 20038 | 20923 | -2960 | -1534 | 0.194 | 0.648 | 78.7 | 7.55 | 90.5 | 4.93 | 80.1 | 9.96 |
