# Supplementary material for: Effect of Pretreatment on Detection of 37 Pesticide Residues in Chrysanthemum indicum
Source: J Anal Methods Chem. 2021 Dec 9;2021:8854025. doi: 10.1155/2021/8854025 (PMC8677409; doi:10.1155/2021/8854025)
Supplement: Supplementary Materials — Some figures and tables are included in the supplementary file. [file 8854025.f1.zip › 8854025.f1/Table 4. Linearity (expressed as R2), limit of quantification (LOQ), and three levels of accuracy of QuEChERS methods.docx]

| Table 4. Linearity (expressed as R2), limit of quantification (LOQ), and three levels of accuracy (expressed as recovery; *: 0.4 mg/kg, **: 2 mg/kg, ***:10mg/kg) of QuEChERS methods | | | | | | | | | | | | | | |
| --- | --- | --- | --- | --- | --- | --- | --- | --- | --- | --- | --- | --- | --- | --- |
| Name | Regression equation | R2 | 95% confidence interval of slope | | 95% confidence interval of intercept | | LOD(mg/kg) | LOQ(mg/kg) | * Recovery/% | * RSD/% | ** Recovery/% | ** RSD/% | *** Recovery/% | *** RSD/% |
|  |  |  | toplimit | lower limit | toplimit | lower limit |  |  |  |  |  |  |  |  |
| α-BHC | y = 19.1x + 181 | 1.00 | 18.8 | 19.4 | -277 | -641 | 0.372 | 1.24 | 90.3 | 3.34 | 100 | 1.20 | 102 | 1.30 |
| γ-BHC | y = 15.4x + 2083 | 1.00 | 14.9 | 16.0 | -829 | -3338 | 0.547 | 1.82 | 106 | 3.66 | 106 | 6.33 | 109 | 3.29 |
| Heptachlor epoxide | y = 11.5x - 808 | 0.999 | 11.2 | 11.8 | -1440 | -176 | 0.717 | 2.39 | 92.1 | 4.38 | 96.4 | 5.23 | 113 | 5.20 |
| Aldrin | y = 24.3x + 41368 | 0.999 | 23.0 | 25.8 | 37639 | 45096 | 0.388 | 1.29 | 86.1 | 8.31 | 112 | 2.30 | 98.7 | 0.66 |
| Alachlor | y = 44.6x - 1312 | 0.999 | 43.8 | 45.3 | -2697 | 72.2 | 0.201 | 0.671 | 114 | 1.40 | 105 | 2.47 | 103.11 | 0.60 |
| β-BHC | y = 15.2x + 6188 | 1.00 | 14.8 | 15.6 | 5271 | 7104 | 0.450 | 1.50 | 94.6 | 9.17 | 100 | 1.28 | 117 | 6.85 |
| δ-BHC | y = 4.99x + 4522 | 1.00 | 4.85 | 5.13 | 4231 | 4812 | 0.849 | 2.83 | 105 | 3.86 | 107 | 2.90 | 119 | 9.18 |
| Heptachlor exo-epoxide | y = 7.96x + 4556 | 0.981 | 5.93 | 10.0 | 870 | 8242 | 0.530 | 1.77 | 107 | 8.19 | 94.2 | 1.27 | 98.1 | 1.04 |
| Pendimethalin | y = 1.65x + 2858 | 0.998 | 1.52 | 1.77 | 2580 | 3136 | 4.21 | 14.00 | 118 | 9.23 | 97.1 | 5.61 | 109 | 1.32 |
| α-Endosulfan | y = 5.44x - 479 | 1.00 | 5.31 | 5.56 | -719 | -238 | 1.92 | 6.41 | 76.0 | 1.90 | 92.8 | 3.73 | 96.8 | 1.99 |
| p,p'-DDE | y = 29.8x - 458 | 0.999 | 29.3 | 30.3 | -1306 | 390 | 0.349 | 1.16 | 81.2 | 3.74 | 100 | 1.7 | 98.2 | 2.70 |
| Dieldrin | y = 3.13x + 5834 | 0.99 | 2.54 | 3.73 | 5276 | 6391 | 3.26 | 10.90 | 80.0 | 1.97 | 81.5 | 10.6 | 98.7 | 3.66 |
| Endrin | y = 5.73x + 2357 | 0.999 | 5.53 | 5.93 | 1917 | 2798 | 1.81 | 6.02 | 92.9 | 4.68 | 100 | 1.21 | 110 | 2.35 |
| m,p'-DDD | y = 35.7x - 421 | 0.999 | 35.1 | 36.3 | -1502 | 660 | 0.325 | 1.08 | 101 | 1.70 | 103 | 1.28 | 104 | 4.82 |
| β-Endosulfan | y = 4.49x - 422 | 0.999 | 4.33 | 4.64 | -758 | -86.9 | 2.68 | 8.93 | 88.0 | 3.66 | 96.0 | 3.21 | 104 | 5.43 |
| Endosulfan sulfate | y = 23.5x + 252 | 1.00 | 22.8 | 24.1 | -1178 | 1681 | 2.59 | 8.62 | 99.3 | 4.00 | 96.6 | 8.49 | 101 | 8.98 |
| Tetradiphon | y = 8.4509x + 1624.3 | 0.999 | 7.15 | 9.75 | -1515 | 4763 | 2.04 | 6.81 | 117 | 1.80 | 108 | 1.35 | 115 | 3.58 |
| o,o,o-Triethylphosphorothioate | y = 37.496x + 11278 | 0.999 | 36.7 | 38.3 | 9768 | 12788 | 0.064 | 0.215 | 105 | 2.09 | 106 | 4.56 | 105 | 3.27 |
| Thionazin | y = 42.981x + 383392 | 1.00 | 42.0 | 44.6 | 379080 | 387703 | 0.048 | 0.160 | 106 | 0.910 | 109 | 2.95 | 93.6 | 2.08 |
| Phorate | y = 71.11x - 1369.7 | 1.00 | 69.4 | 72.8 | -4579 | 1839 | 0.079 | 0.264 | 98 | 4.15 | 106 | 1.94 | 100 | 2.29 |
| Sulfotep | y = 40.054x + 29838 | 0.998 | 37.0 | 43.1 | 24392 | 35285 | 0.148 | 0.493 | 109 | 6.13 | 108 | 2.53 | 109 | 7.14 |
| Pentachloronitrobenzene | y = 13.338x + 245.76 | 0.999 | 13.0 | 13.6 | -297 | -961 | 0.471 | 1.57 | 92.6 | 5.87 | 104 | 1.84 | 105 | 4.49 |
| Diazinone | y = 40.265x + 256.66 | 0.999 | 39.4 | 41.1 | -1364 | 1878 | 0.139 | 0.465 | 102 | 1.35 | 120 | 2.60 | 104 | 0.160 |
| Disulfoton | y = 0.2946x + 779.07 | 0.997 | 0.195 | 0.394 | 552 | 1006 | 0.534 | 1.78 | 80.8 | 5.13 | 97.1 | 0.92 | 100 | 4.79 |
| Dimethoate | y = 16.971x + 8135.8 | 0.999 | 16.3 | 17.7 | 6707 | 9565 | 0.452 | 1.51 | 84.7 | 7.73 | 109 | 3.56 | 97.0 | 5.16 |
| Ronnel | y = 26.389x - 202.92 | 1.00 | 25.9 | 26.9 | -1117 | -711 | 0.246 | 0.821 | 94.4 | 4.14 | 95.4 | 3.58 | 111 | 1.61 |
| Metalaxyl | y = 32.963x + 42802 | 1.00 | 31.6 | 34.3 | 39717 | 45887 | 0.216 | 0.72 | 108 | 7.70 | 100 | 1.13 | 104 | 0.47 |
| Chlorpyrifos | y = 39.932x + 52808 | 1.00 | 38.7 | 41.4 | 49546 | 56070 | 0.179 | 0.597 | 91.3 | 5.40 | 89.5 | 1.50 | 111 | 4.04 |
| Methyl parathion | y = 20.319x - 18680 | 0.995 | 17.7 | 22.9 | -26195 | -11164 | 0.293 | 0.976 | 79.0 | 3.32 | 90.3 | 2.60 | 91.3 | 1.84 |
| Fenthion | y = 40.707x + 3872.4 | 1.00 | 39.9 | 41.4 | 2315 | 5430 | 0.201 | 0.671 | 84.8 | 6.2 | 100 | 1.69 | 109 | 2.38 |
| Bromophos | y = 15.2x + 16587 | 0.999 | 14.4 | 16.0 | 14413 | 18761 | 0.462 | 1.54 | 96.3 | 6.83 | 117 | 4.88 | 118 | 0.780 |
| Parathion | y = 23.97x + 2324.1 | 0.999 | 23.2 | 24.7 | 933 | 3715 | 0.334 | 1.11 | 89.6 | 9.49 | 101 | 9.28 | 117 | 3.43 |
| Quinalphos | y = 28.495x - 1380.7 | 1.00 | 27.8 | 29.2 | -2687 | -74.0 | 0.295 | 0.983 | 85.2 | 3.22 | 110 | 3.59 | 110 | 2.46 |
| Procymidone | y = 0.1694x + 752.21 | 0.997 | 0.154 | 0.185 | 710 | 795 | 0.626 | 2.09 | 95.1 | 6.38 | 118 | 1.76 | 94.7 | 2.07 |
| Profenofos | y = 0.165x + 426.62 | 0.998 | 0.152 | 0.178 | 407 | 446 | 0.579 | 1.93 | 102 | 7.26 | 98.1 | 7.76 | 87.0 | 3.69 |
| Famphur | y = 5.4014x + 581.23 | 0.999 | 5.21 | 5.59 | 141 | 1021 | 0.816 | 2.72 | 96.6 | 5.74 | 99.1 | 2.21 | 105 | 3.67 |
| Quizalofop ethyl | y = 6.1294x - 93.28 | 0.999 | 5.93 | 6.33 | -475 | 289 | 1.80 | 6.00 | 106 | 0.280 | 111 | 3.32 | 115 | 6.41 |
