# Supplementary figures and images for: Effect of Pretreatment on Detection of 37 Pesticide Residues in Chrysanthemum indicum
Source: J Anal Methods Chem. 2021 Dec 9;2021:8854025. doi: 10.1155/2021/8854025 (PMC8677409; doi:10.1155/2021/8854025)

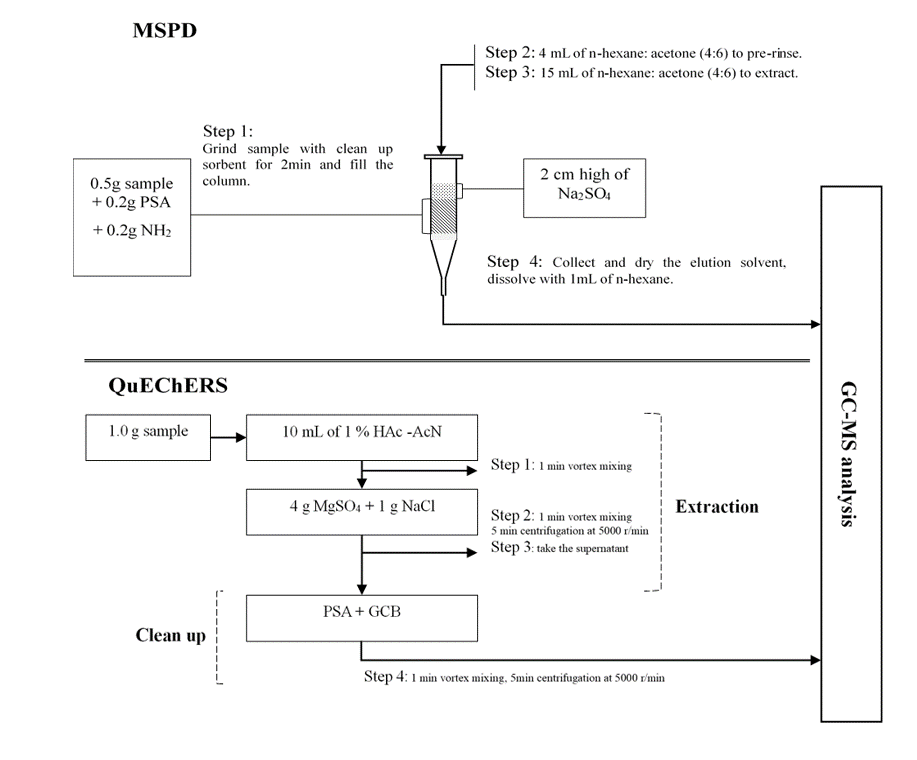


Supplementary Fig.1 Experimental procedure for MSPD and QuEChERS methods.

Supplement: Supplementary Materials — Some figures and tables are included in the supplementary file. [file 8854025.f1.zip › 8854025.f1/Supplementary Fig.1 Experimental procedure for MSPD and QuEChERS methods. (1).docx]
